# Improved Resolution of Influenza Vaccination Responses With High‐Throughput Live Virus Microneutralisation

**DOI:** 10.1111/irv.70140

**Published:** 2025-08-14

**Authors:** Lorin Adams, Phoebe Stevenson‐Leggett, Jia Le Lee, James Bazire, Giulia Dowgier, Agnieszka Hobbs, Chloë Roustan, Annabel Borg, Christine Carr, Silvia Innocentin, Louise M. C. Webb, Callie Smith, Philip Bawumia, Nicola Lewis, Nicola O'Reilly, Svend Kjaer, Michelle A. Linterman, Ruth Harvey, Mary Y. Wu, Edward J. Carr

**Affiliations:** ^1^ Worldwide Influenza Centre The Francis Crick Institute London UK; ^2^ COVID Surveillance Unit The Francis Crick Institute London UK; ^3^ Immunology Program Babraham Institute Cambridge UK; ^4^ The Francis Crick Institute London UK; ^5^ UCL Centre for Kidney & Bladder Health Royal Free Hospital London UK

**Keywords:** haemagglutinin inhibition assays, influenza, live virus microneutralisation, vaccine

## Abstract

**Background:**

Influenza remains a significant threat to human and animal health. Assessing serological protection against influenza has relied upon haemagglutinin inhibition (HAI) assays, which are used to gauge existing immune landscapes, seasonal vaccine decisions and in systems vaccinology studies. HAI assays were first described in the 1940s. Here, we adapt our high‐throughput live virus microneutralisation (LV‐N) assay for SARS‐CoV‐2, benchmark against HAI assays, and report serological vaccine responsiveness in a cohort of older (> 65 yo) community dwelling adults.

**Methods:**

Influenza‐specific antibody responses were assessed in 73 individuals, before and after receipt of the adjuvanted 2021–22 Northern Hemisphere quadrivalent vaccine. We performed both HAI and LV‐N assays against all four viruses represented in the vaccine [A/Cambodia/e0826360/2020 (H3N2), IVR‐215 (A/Victoria/2570/2019‐like) (H1N1)pdm09, B/Phuket/3073/2013 (B/Yamagata lineage), B/Washington/02/2019 (B/Victoria lineage)], using sera drawn before vaccination [range: d‐82 to d‐5], and days 7 [d6–10] and 181 [d156–200] after vaccination. We compared serological responses within each assay and between assays.

**Results:**

Both the traditional HAI assay and our high‐throughput live virus microneutralisation identified vaccine‐induced boosts in antibody titres. We found population‐level concordance between the two assays (Spearman's correlation coefficient range 0.49–0.88; all *p* ≤ 1.4 × 10^−5^). The improved granularity of microneutralisation was better able to estimate fold changes of responses and quantify the inhibitory effect of pre‐existing antibody.

**Conclusions:**

Our high‐throughput method offers an alternative approach to assess influenza‐specific serological responses with improved resolution, with the potential to improve the annual assessment of existing antibody landscapes, to improve new vaccine strain evaluation, and to offer a step‐change in systems vaccinology, and a facet of laboratory‐based pandemic preparedness.

## 1 Introduction

Influenza causes an estimated 389,000 deaths a year [1]. Whilst annually updated multi‐valent vaccines reduce this burden, rapid, granular evaluation of neutralising antibody responses could further minimise this in several ways. Firstly, by facilitating refined yearly strain selection through high‐resolution quantification of the pre‐existing antibody landscape. Secondly, by prompt, robust comparison between vaccine platforms, both existing (protein, adjuvanted protein, live‐attenuated virus or split virion) and upcoming (mRNA, and others). Thirdly, by providing a continuous response variable for systems vaccinology studies to better leverage multi‐modal high‐dimensional and longitudinal datasets, themselves generated at significant cost and resource. Finally, by allowing timely re‐evaluation of human population immunity when an antigenically shifted influenza virus emerges, such as H5N1.

Currently, the haemagglutinin inhibition (HAI) assay is used to evaluate existing population‐level immunity to guide annual vaccine strain selection, to confirm immunogenicity of these updated preparations, and as a correlate of protection. HAI assays are largely unchanged since first described in the 1940s [2, 3]: they are scalable, require minimal specialist laboratory equipment, but do not directly measure neutralising antibody, a critical effector arm of immunity. Virus will agglutinate red blood cells, naturally decorated with sialic acid, the ligand for influenza's HA. Agglutination is inhibited by sera containing HA‐specific antibodies. Live‐virus neutralisation assays for influenza have existed for decades [4], but have to date not been capable of displacing the HAI assay, largely due to limitations in the number of sera processed and their relative complexity.

In this study, we performed a head‐to‐head comparison of paired HAI and our newly developed high‐throughput live‐virus microneutralisation (LV‐N) assay. Adapting our live virus SARS‐CoV‐2 approach, the assay pipeline retains a similar throughput of ~1000–1500 sera for three strains, or smaller batches of sera against more strains to produce approximately 4500 total neutralisation results per week. This work demonstrates that large‐scale microneutralisation assays of four influenza strains are both feasible and offer enhanced serological resolution over HAIs.

## 2 Methods

### 2.1 Study Design, Serum Collection and Ethics

The immune responses to vaccines in older persons study (AgeVax) enrolled community‐dwelling older adults already enrolled within the NIHR Cambridge BioResource to assess their response to the seasonal influenza vaccine. AgeVax was approved by the Yorkshire & The Humber—South Yorkshire Research Ethics Committee (IRAS 277259, REC 20/YH/0101) and sponsored by the Babraham Institute, Cambridge. Healthy participants were enrolled if they were planning to receive the seasonal influenza vaccine as part of their routine care (in the UK, influenza vaccine is offered to all > 65 yo), were able to attend for consenting and venepuncture, and carried at least one allele of HLADR*0701, HLADR*0401 or HLADR*1101 (determined by single nucleotide polymorphism typing using the UK Biobank v2.1 Axiom array). Controlled comorbidities such as hypertension or hypercholesterolaemia were not exclusion criteria. Concomitant medications and comorbidities are not available for research purposes.

Venepuncture was performed up to 82 days pre‐vaccination and on days 6–10 and 156–200 post‐vaccination. All participants received the adjuvanted quadrivalent influenza vaccine (Seqirus UK Ltd.) via intramuscular injection. Blood was collected into silica‐coated serum tubes and centrifuged at 2000 rpm for 5 min and stored in aliquots at −80°C. These sera were provided to the Francis Crick Institute for influenza assays described below.

### 2.2 Cells and Viruses

Madin–Darby Canine Kidney (MDCK) cells expressing the SIAT1 gene (MDCK‐SIAT1 [5]) were maintained under selection in DMEM containing 1 mg/mL Geneticin (G418) Sulphate (Stratech Scientific APE2513) and 1% penicillin/streptomycin (Sigma‐Aldrich). Cells were seeded in DMEM containing 1% Penicillin/Streptomycin into 384‐well cell culture microplates (Griener Bio‐One Ltd) 18–20 h prior to use in live‐virus microneutralisation assays.

All influenza virus isolates used in this study were propagated in the allantoic cavity of 10‐day‐old embryonated hens' eggs at 35°C for 48 h.

### 2.3 HAI Assays for Influenza

Haemagglutination and HAI assays were performed according to standard methods using suspensions of guinea pig RBCs (1.0% v/v) for A/Cambodia/e0826360/2020 (H3N2) viruses and turkey RBCs (0.75% v/v) for IVR‐215 (A/Victoria/2570/2019‐like) (H1N1)pdm09, and B viruses (B/Phuket/3073/2013 (B/Yamagata lineage), B/Washington/02/2019 (B/Victoria lineage)) with all serum samples pre‐treated with receptor‐destroying enzyme (RDE) from Vibrio cholera [6]. Four haemagglutination units were used in all HAI assays. For A(H3N2) viruses, haemagglutination and HAI assays were conducted in the presence of 20 nM oseltamivir carboxylate [7]. These four viruses matched the 2021–22 Northern hemisphere quadrivalent vaccine recommendation.

### 2.4 Live‐Virus Microneutralisation Assays for Influenza

To perform LV‐N assays for influenza, we adapted our existing approach for SARS‐CoV‐2 neutralisation [8, 9, 10]. MDCK‐SIAT1 cells at 80% confluency were infected with selected influenza isolates in 384‐well format, in the presence of 10‐fold serial dilutions of participant serum samples, diluted in DMEM containing 1% Penicillin/Streptomycin. Trypsin was not included in the media to limit rounds of reinfection. Prior to running the assay, each virus was titrated in 384‐well assay format to ascertain the correct dilution to achieve an infection level of approximately 50% after 24 h incubation at 37°C. At the endpoint of the assay, cells were fixed using 4% formaldehyde (v/v), permeabilised with 0.2% TritonX‐100 with 3% BSA in PBS (v/v) and stained for influenza nucleoprotein (NP) using Biotin‐labelled clone 2‐8C antibody [11] produced in‐house in conjunction with an Alexa488‐Streptavidin (Invitrogen S32354) for influenza A isolates, or the mouse monoclonal B017 (Abcam, ab20711) in conjunction with an Alexa488 conjugated Goat anti‐Mouse secondary (ThermoFisher, A21141) for influenza B isolates. Cellular DNA was stained using DAPI. Whole‐well imaging at 5× magnification was carried out using an Opera Phenix (Perkin Elmer) and fluorescent areas were calculated using the Phenix‐associated software Harmony (Perkin Elmer). At 5× magnification, 90% of each well was captured and encompassed approximately 4000 cells. Virus inhibition by participant serum samples was estimated from (the measured area of infected cells)/(total area occupied by all cells) in each well and then expressed as a percentage of maximal (virus only control wells). Infected cells were identified by the presence of influenza NP staining. The inhibitory profile of each serum sample was estimated by fitting a four‐parameter dose–response curve executed in SciPy. Neutralising antibody titres are reported as the fold dilution of serum required to inhibit 50% of viral replication (IC_50_) and are further annotated if they lie above the quantitative range (> 40,000), below the quantitative range (< 40) but still within the qualitative range (i.e., partial inhibition is observed but a dose–response curve cannot be fitted because it does not sufficiently span the IC_50_), or if they show no inhibition at all.

Viral isolates used in this study: A/Cambodia/e0826360/2020 (H3N2), IVR‐215 (A/Victoria/2570/2019) A(H1N1)pdm09, B/Phuket/3073/2013 (B/Yamagata lineage), B/Washington/02/2019 (B/Victoria lineage).

### 2.5 Data Curation and Analyses

HAI and LV‐N titres were associated with anonymized metadata using R (v 4.2.2) and *tidyverse* (v 1.3.2) [12]. Plots were generated using *ggplot2* (v 3.5.1), with *stat_summary()* to add geometric means or medians, as indicated in the figure legends. To plot HAI titres, sera with no inhibition were re‐coded as 2.5. To plot LV‐N titres, sera with either no inhibition of viral entry, or qualitative inhibition below the quantitative range (40–40,000), or inhibition greater than the quantitative range were re‐coded as 5, 10 or 80,000, respectively. HAI and LV‐N results were compared using two‐tailed paired Wilcoxon tests, as implemented in the *rstatix* package, and resulting P values were plotted using *ggpubr::geom_bracket()* and *ggtext::geom_richtext()*. Bias‐corrected 95% confidence intervals for fold changes in HAI and LV‐N were calculated from 1000 bootstraps generated with the *infer* package [13]. For correlation between HAI and LV‐N, Spearman's correlation was used without censoring data above or below the quantitative ranges of HAI or LV‐N. Anonymised data and R code are freely available via GitHub: https://github.com/EdjCarr/AgeVax_HAI_LVN

## 3 Results

**FIGURE 1:**
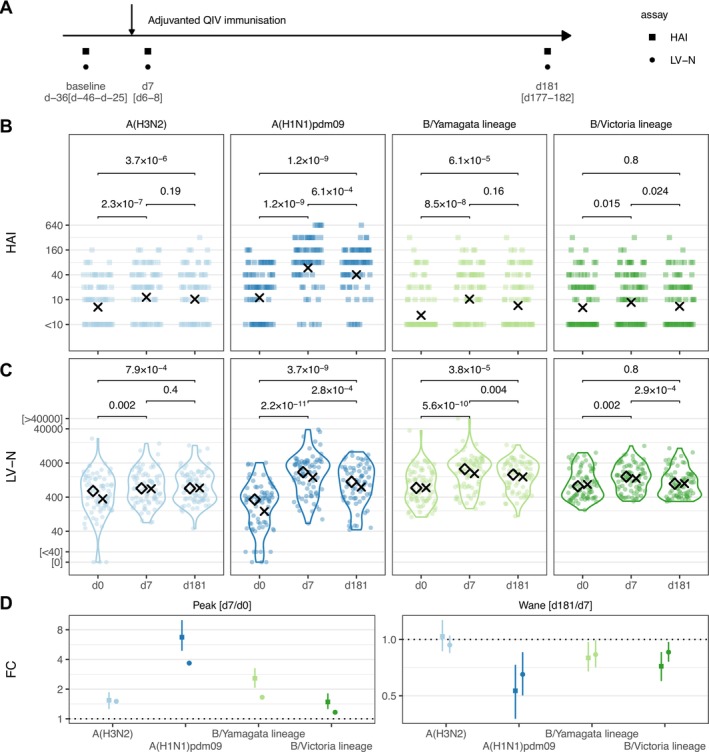
Serological vaccination responses quantified by haemagglutinin inhibition and high‐throughput live influenza neutralisation assays in the AgeVax study. (A) AgeVax study design 73 individuals > 65 yo were vaccinated with adjuvanted quadrivalent influenza vaccine in the 2021–22 Northern Hemisphere season. The median and interquartile ranges of the interval in days between serum draws and vaccination is shown. (B & C) Haemagglutinin inhibition assay titres (HAI, B) or live‐virus microneutralisation assay titres (LV‐N, C), expressed as the reciprocal of the dilution at which 50% of virus infection is inhibited (IC_50_), at baseline (d0) and Days 7 and 181 after vaccination (d7 & d181, respectively) for the flu viruses listed. (D) Log_2_ fold changes for peak (d7/d0) and wane (d181/d7) response as measured by HAI or LV‐N (plotted as squares or circles respectively). Mean and bootstrapped 95% confidence intervals are shown. In (B & C), crosses indicate geometric means, and *p* values from two‐tailed paired Wilcoxon signed rank tests are shown. In (C), diamonds indicate medians. Influenza viruses A/Cambodia/e0826360/2020 (H3N2), IVR‐215 (A/Victoria/2570/2019‐like) (H1N1)pdm09, B/Phuket/3073/2013 (B/Yamagata lineage), B/Washington/02/2019 (B/Victoria lineage) were used.

To compare HAI and LV‐N assays, we used sera from the AgeVax study, an observational study of influenza vaccine responses in 73 community‐dwelling older adults (> 65 yo), with venepuncture on days 0 (range: d‐82 to d‐5), 7 (d6–10) and 181 (d156–200) post‐vaccination (Table 1 , Figure 1A).

**TABLE 1:** Demographic summary of the AgeVax study.

Characteristic	AgeVax, *N* = 73^a^
Age	70.0 [68.0–74.0]
Sex	
F	35 (48%)
M	38 (52%)

^a^ Median [25%–75%]; *n* (%).

We first confirmed increased antibody titres for all four influenza strains at d7 after administration of the 2021–22 vaccine using HAI (d7 vs. d0 Wilcoxon *p* < 0.015 for all strains, Figure 1B). Between paired sera, we found waning from d7 to d181 for two of the four immunised strains (A(H3N2) *p* = 0.19; A(H1N1)pdm09 *p* = 6.1 × 10^−4^; B/Yamagata lineage *p* = 0.16; B/Victoria lineage *p* = 0.024, Figure 1B). We next repeated this immunogenicity assessment using our newly developed LV‐N assay (Figure 1C). The boosting effect of vaccination was, once again, clear (d7 vs. d0 *p* < 0.002 for all strains, Figure 1C). With the LV‐N assay, three of the four immunised strains showed significant waning between d7 and d181 A(H3N1) *p* = 0.4; A(H1N1)pdm09 *p* = 2.8 × 10^−4^; B/Yamagata lineage *p* = 0.004; B/Victoria lineage *p* = 2.9 × 10^−4^. The quantitative nature of the LV‐N assays offered improved resolution of the fold‐change of the acute response (in the AgeVax study, the fold‐change in titres d7/d0), with geometric mean fold‐changes [95% confidence intervals] for LV‐N estimating smaller reductions in boosts with less uncertainty between these visits when compared with HAI (Figure 1D). Furthermore, the waning (d181/d7) of B/Yamagata lineage reached statistical significance only within the LV‐N dataset (Figures 1B,C).

**FIGURE 2:**
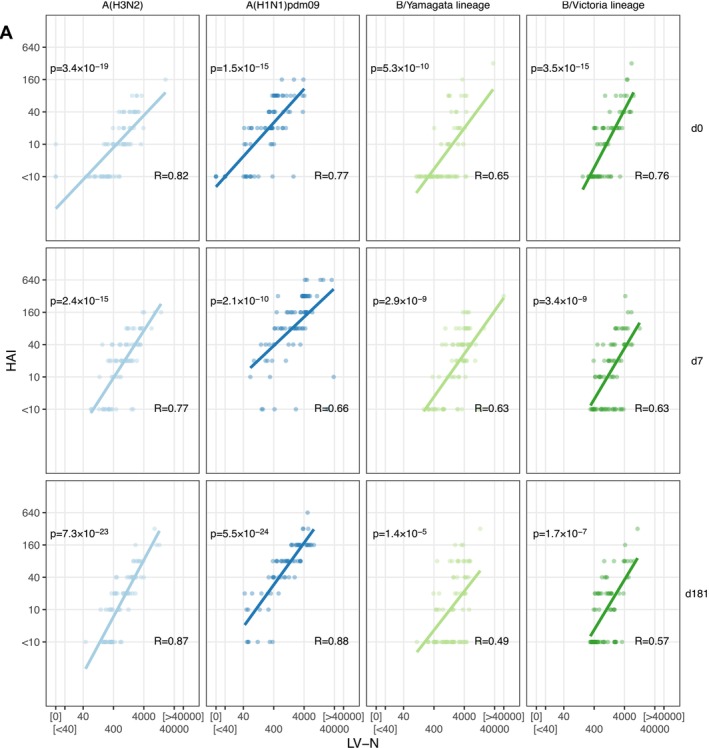
Comparison of haemagglutinin inhibition and high‐throughput live influenza neutralisation assays. (A) Correlation between HAI and LV‐N for each flu virus tested, at each venepuncture. Spearman correlation coefficients and *p* values are shown, with a fit line from linear regression performed after log2 transformation. Influenza viruses A/Cambodia/e0826360/2020 (H3N2), IVR‐215 (A/Victoria/2570/2019‐like) (H1N1)pdm09, B/Phuket/3073/2013 (B/Yamagata lineage), B/Washington/02/2019 (B/Victoria lineage) were used.

Next, we plotted pairwise comparisons between HAI and LV‐N for each strain at each of the three venepuncture visits (Figure 2A). Overall, the two assays showed a strong positive correlation according to their Spearman's correlation coefficients. Considering differences between strains, B/Yamagata showed relatively weaker correlations than the other three strains. Considering differences over three timepoints, the between‐assay correlation for B/Victoria lineage was weakest at d181, but no other strains showed marked differences over time.

**FIGURE 3:**
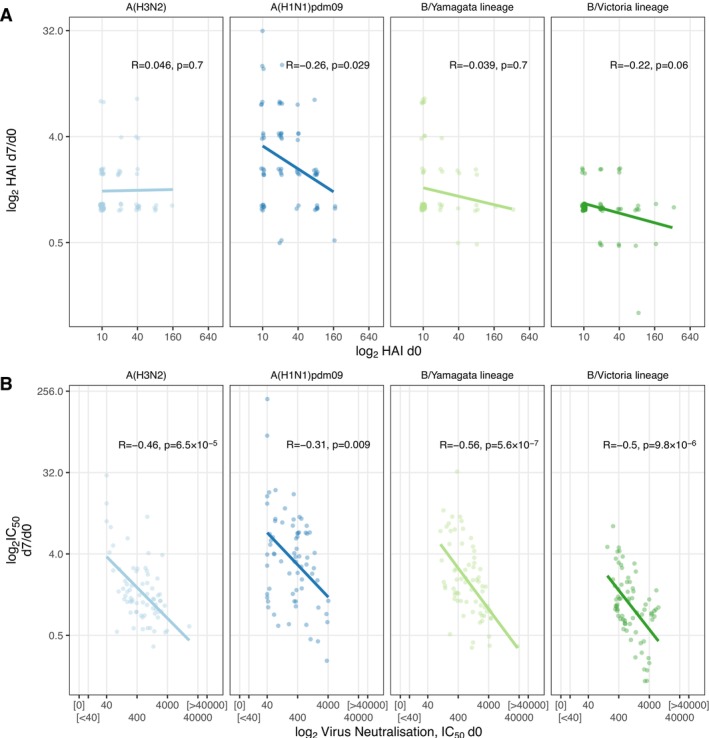
Pre‐existing antibody inhibits subsequent vaccine responses. (A & B) Log_2_ fold changes of peak titres (d7/d0) plotted against d0 titres for HAI (A) or LV‐N (B). Spearman correlation coefficients and *p* values are shown, as is the fit line from linear regression performed after log2 transformation. In (A & B) to avoid inflating fold changes, values below the quantitative range of either assay were rounded to the bottom of the relevant quantitative range; this adjustment was performed before plotting and statistical testing. Influenza viruses A/Cambodia/e0826360/2020 (H3N2), IVR‐215 (A/Victoria/2570/2019‐like) (H1N1)pdm09, B/Phuket/3073/2013 (B/Yamagata lineage), B/Washington/02/2019 (B/Victoria lineage) were used.

Despite the high concordance between assays at a population level, we noted that for a given HAI titre, the range of corresponding LV‐N results was large: for example, an HAI titre of 20 at baseline (d0, *n* = 19) corresponded to an LV‐N range of 43–1004 for influenza A(H1N1)pdm09. This 23‐fold difference suggests that immunologically important differences in vaccine responses were aggregated into discrete HAI values. To address the question of whether LV‐N offered improved sensitivity for biologically relevant heterogeneity over HAI, we returned to the observation that pre‐existing antibody limits the fold induction of antibody after vaccination [14]. Using HAI, we found that we could observe this negative relationship between higher baseline titres and lower fold changes (d7/d0) for only A(H1N1)pdm09 (*p* = 0.029), with the other strains showing non‐significant trends (Figure 3A). Repeating this comparison with LV‐N, we found moderately strong negative correlations between pre‐existing neutralising antibody and peak fold change (d7/d0) for all variants (all *p* < 0.009, Figure 3B).

## 4 Discussion

Here, we have described an approach for high‐throughput live virus microneutralisation of influenza and benchmarked this approach against HAI. LV‐N outperforms HAI in two ways. Firstly, the capacity of LV‐N to assay thousands of sera per week using 384 well plates and workflow automation greatly exceeds the capacity of a laboratory scientist conducting manual HAIs. Secondly, the LV‐N assay reports IC_50_ across a large quantitative range, whereas HAI aggregates neutralisation into dilution factors, with the risk of obfuscating smaller but important quantitative differences (Figure 3). This obfuscating effect is particularly relevant for systems vaccinology in which antibody responses coarsely measured with HAI are compared to large, quantitative multi‐modal omic datasets. The quantitative nature of LV‐N promises to unlock new potentials in systems vaccination, examining peak and waned responses, both within and between vaccine formulations.

There are some limitations to our study. This single centre study enrolled older healthy adults, likely to mount a memory response, recalling both previously haemagglutinin‐experienced B cells and recruiting naive B cells. It is plausible that a primary influenza vaccine response might be alternatively performant between these assays, particularly if significant IgM is produced, likely to perform well at HAI. Additionally, our live‐virus microneutralisation approach reflects serological neutralisation of both HA and neuraminidase during viral entry, whereas HAI does not assay NA‐directed neutralisation. Furthermore, HA2 subunit, or HA‐stalk, directed neutralising antibodies might only be detected in LV‐N and missed by HAI (a metric of the HA1 subunit binding to sialic acid), and therefore some of the differences in Figure 3 might reflect these different pools of antibodies, as well as granular quantitation. We have not compared inter‐batch variation between HAI and LV‐N, as the entirety of the serum set was processed for each strain in a single batch. Our SARS‐CoV‐2 live virus assay gives an indication of the magnitude of the variability between LV‐N batches performed months apart: we observe < 2 fold variation between assay batches over time. For example, sotrovimab EC_50_ against BA.1 was measured as 399.1 (95% confidence intervals 308.3–516.7) [15] and 291.8 (95% confidence intervals 269.4–314.5) [16], in experimental runs separated by approximately 4 months. Other implementations of microneutralisation assays use alternative readouts for infection: for example, the WHO protocol uses an ELISA for nucleoprotein on fixed MDCK cells (reporting the lowest dilution at which the OD405 is below the midpoint of OD405 of virus‐only and cell‐only controls) [17], and a recently described next‐generation sequencing assay testing infectivity/neutralisation of many DNA‐barcoded viruses in parallel [18]. Whilst, we have not directly compared with these methods, the DNA‐barcoding approach can only find HA‐related neutralisation, and the WHO neutralisation assay reports discrete rather than continuous data.

## 5 Conclusion

In conclusion, we have shown that high‐throughput live‐virus microneutralisation assays are non‐inferior to HAI with strong correlations at population levels. Further, we find that the continuous titre values returned by LV‐N allow for finer dissection of vaccine responses that are obfuscated by HAI. High‐throughput influenza LV‐N therefore has the potential to be a catalyst for rapid, robust assessment of existing antibody landscapes, new vaccine strain formulations, a step change in systems vaccinology, and a facet of laboratory‐based pandemic preparedness.

## Author Contributions

**Lorin Adams:** conceptualization, investigation, methodology. **Phoebe Stevenson‐Leggett:** conceptualization, investigation, methodology. **Jia Le Lee:** project administration, methodology, investigation. **James Bazire:** methodology, investigation. **Giulia Dowgier:** methodology, investigation. **Agnieszka Hobbs:** investigation, methodology. **Chloë Roustan:** resources. **Annabel Borg:** resources. **Christine Carr:** resources. **Silvia Innocentin:** methodology, investigation. **Louise Webb MC:** methodology, investigation. **Callie Smith:** resources. **Philip Bawumia:** resources. **Nicola Lewis:** resources, supervision. **Nicola O’Reilly:** resources, supervision. **Svend Kjaer:** resources, supervision. **Michelle Linterman A:** conceptualization, funding acquisition, supervision, writing – review and editing. **Ruth Harvey:** conceptualization, funding acquisition, investigation, supervision, writing – review and editing. **Mary Wu Y:** conceptualization, funding acquisition, investigation, supervision, writing – review and editing. **Edward Carr J:** conceptualization, data curation, formal analysis, visualization, writing – original draft, writing – review and editing.

## Conflicts of Interest

M.A.L. is part of the GSK Immunology Network and reports funding from GSK outside this study. The other authors declare no potential conflicts of interest.

## Data Availability

Anonymised data and R code are freely available via github: https://github.com/EdjCarr/AgeVax_HAI_LVN.
